# 10-Ethyl-10*H*-phenothia­zine-3-carbaldehyde

**DOI:** 10.1107/S1600536811047799

**Published:** 2011-11-19

**Authors:** Dao-Hui Yu, Jian-Qing Wang, Lin Kong, Zhao-di Liu

**Affiliations:** a Department of Chemistry, Anhui University, Hefei 230039, People’s Republic of China, and Key Laboratory of Functional Inorganic Materials, Chemistry, Hefei 230039, People’s Republic of China

## Abstract

In the title mol­ecule, C_15_H_13_NOS, the two benzene rings of the tricyclic fused-ring system are inclined at 21.1 (1)°. In the crystal, weak C—H⋯O hydrogen bonds link the mol­ecules into chains along [001]. The crystal packing also exhibits π–π inter­actions with a distance of 3.801 (5) Å between the centroids of the benzene rings of neighbouring mol­ecules.

## Related literature

For related structures, see: Chu & Van der Helm (1975 ▶); Hdii *et al.* (1998 ▶); Li *et al.* (2009*a* ▶,*b* ▶).
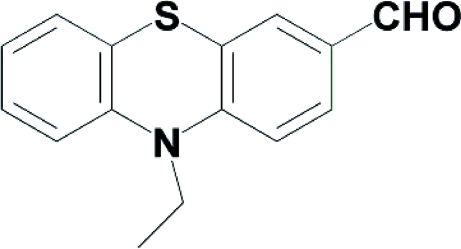


## Experimental

### 

#### Crystal data

C_15_H_13_NOS
*M* *_r_* = 255.32
Orthorhombic, 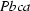

*a* = 8.0867 (1) Å
*b* = 15.3271 (3) Å
*c* = 20.3369 (4) Å
*V* = 2520.67 (8) Å^3^
*Z* = 8
Mo *K*α radiation
μ = 0.24 mm^−1^
*T* = 296 K
0.20 × 0.10 × 0.10 mm

#### Data collection

Bruker SMART APEX diffractometer
Absorption correction: multi-scan (*SADABS*; Sheldrick, 1996 ▶) *T*_min_ = 0.953, *T*_max_ = 0.976
17210 measured reflections
2225 independent reflections
1909 reflections with *I* > 2σ(*I*)
*R*_int_ = 0.028

#### Refinement

*R*[*F* ^2^ > 2σ(*F* ^2^)] = 0.041
*wR*(*F* ^2^) = 0.109
*S* = 1.08
2225 reflections
167 parameters
H atoms treated by a mixture of independent and constrained refinement
Δρ_max_ = 0.40 e Å^−3^
Δρ_min_ = −0.42 e Å^−3^

### 

Data collection: *SMART* (Bruker, 2002 ▶); cell refinement: *SAINT* (Bruker, 2002 ▶); data reduction: *SAINT*; program(s) used to solve structure: *SHELXS97* (Sheldrick, 2008 ▶); program(s) used to refine structure: *SHELXL97* (Sheldrick, 2008 ▶); molecular graphics: *SHELXTL* (Sheldrick, 2008 ▶); software used to prepare material for publication: *SHELXTL*.

## Supplementary Material

Crystal structure: contains datablock(s) I, global. DOI: 10.1107/S1600536811047799/cv5184sup1.cif

Structure factors: contains datablock(s) I. DOI: 10.1107/S1600536811047799/cv5184Isup2.hkl

Supplementary material file. DOI: 10.1107/S1600536811047799/cv5184Isup3.cml

Additional supplementary materials:  crystallographic information; 3D view; checkCIF report

## Figures and Tables

**Table 1:** Hydrogen-bond geometry (Å, °)

*D*—H⋯*A*	*D*—H	H⋯*A*	*D*⋯*A*	*D*—H⋯*A*
C14—H14*A*⋯O1^i^	0.97	2.64	3.563 (3)	158

Symmetry code: (i) 
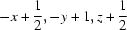
.

**Fig. 1.**
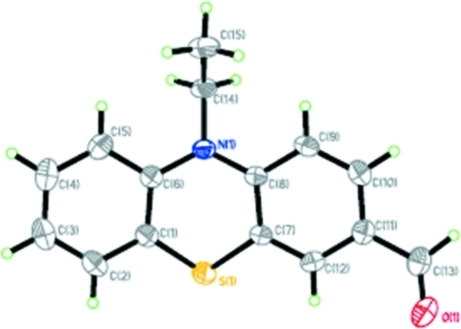
: The molecular structure of the title molecule with 50% probability displacement ellipsoids.

## supplementary crystallographic information

### Comment

The title compound (I) is often used as intermediate in the synthesis of organic compounds with optical properties (Li *et al.*, 2009**a*, b*). Herewith we present its crystal structure.

In (I) (Fig.1), two benzene rings form the dihedral angles of 10.0 (8)° and 12.0 (8)°, respectively, with the thiomorpholine mean plane. The folding of the molecule is characterized by dihedral angle formed by two benzene rings, which is 21.1 (1) °. In the related compounds, 10-ethylphenothiazine (Chu *et al.*, 1975) and 10-ethyl-3-nitrophenothiazine (Hdii *et al.*, 1998), the corresponding dihedral angle is 44.9 (1) and 22.8 (1) °, respectively, showing that any substitution added to benzene ring flattens the tricycle. This tendency also observed in the structure of (*E*)-3-(10-ethyl-10*H*-phenothiazin-3-yl)acrylic acid (Li *et al.*, 2009*b*), where these dihedral angles in two independent molecules are 25.3 (9) ° and 29.8 (8) °, respectively. The ethyl group in (I) is almost orthogonal to the thiazine ring, the torsion angle C6–N1—Cl4–C15 is 85.6 (1)°. While in 10-ethylphenothiazine (Chu *et al.*, 1975), the corresponding angle is 146.1 (4) °, and in 10-ethyl-3-nitrophenothiazine (Hon *et al.*, 1998) this angle is -84.9 (2)°. The aldehyde group is almost coplanar with its attached phenyl ring, the torsion angle C12–C11–C13–O1 being -2.59 °.

In the crystal, weak intermolecular C—H···O hydrogen bonds (Table 1) link molecules into chains along [001]. The crystal packing exhibits π···π interactions with the distance of 3.801 (5) Å between the centroids of benzene rings from the neighbouring molecules.

### Experimental

NaH (4.08 g, 0.17 mol) and DMF (5 ml) were added to a three-necked flask equipped with a magnetic stirrer and a reflux condenser, and then phenothiazine (20.0 g, 0.1 mol), DMF (10 ml) were added dropwisely (about 30 min), refluxed for another 20 min. Then C_2_ H_5_Br (17 ml) was dropped into the mixture and refluxed for 2 h with TLC detection. The pH of the solution was adjusted to acidic with hydrochloric acid then extracted with 500 ml of ethyl acetate, washed three times with distilled water, and dried with anhydrous magnesium sulfate. It was then filtered and concentrated to produce 18.2 g needle crystals in 80% yield.

*N*-ethyl-phenothiazine (11.35 g, 0.05 mol) and DMF (39 ml) were added to a three-necked flask in ice equipped with a magnetic stirrer and a reflux condenser, then POCl_3_ (92 ml) was added dropwisely (about 30 min), the mixture was refluxed for 1 h. Then the mixture was poured into ice to get light yellow solid. The pH of the mixture was adjusted to neutral with NaOH and extracted three times with 150 ml of ethyl acetate. The organic layer was washed with distilled water and then saturated brine. The organic extracts were dried with anhydrous magnesium sulfate. The solvent was removed *in vacuo*. The residue was purified by column chromatography on silica gel with petroleum ether as eluent to give 7.6 g titled compound as a yellow solid in 60% yield. ^1^H NMR (400 MHz, CDCl_3_) 7.569 (s, 1H), 7.157 (t, 1H), 7.097 (d, J = 7.8 Hz, 1H), 6.912 (d, J = 6.4 Hz, 1H), 6.896 (d, J = 6.2 Hz, 1H), 3.974 (q, 2H), 1.447 (t,3*H*). ^13^C NMR (100 MHz). 89, 42.47, 114.40, 115.58, 123.30, 123.56, 124.51, 127.49, 127.59, 128.25, 130.16, 131.04, 189.98.

### Refinement

The methine H atoms was located on a difference map and isotropically refined. All the rest H atoms were placed in geometrically idealized positions (C—H = 0.93 - 0.97 Å) and constrained to ride on their parent atoms, with *U*_iso_(H) = 1.2-1.5 *U*_eq_(C).

### Crystal data

**Table:**

C_15_H_13_NOS	*F*(000) = 1072
*M**_r_* = 255.32	*D*_x_ = 1.346 Mg m^−^^3^
Orthorhombic, *P**b**c**a*	Mo *K*α radiation, λ = 0.71073 Å
Hall symbol: -P 2ac 2ab	Cell parameters from 7387 reflections
*a* = 8.0867 (1) Å	θ = 2.7–27.1°
*b* = 15.3271 (3) Å	µ = 0.24 mm^−^^1^
*c* = 20.3369 (4) Å	*T* = 296 K
*V* = 2520.67 (8) Å^3^	Needle, yellow
*Z* = 8	0.20 × 0.10 × 0.10 mm

### Data collection

**Table:**

Bruker SMART APEX diffractometer	2225 independent reflections
Radiation source: fine-focus sealed tube	1909 reflections with *I* > 2σ(*I*)
graphite	*R*_int_ = 0.028
phi and ω scans	θ_max_ = 25.0°, θ_min_ = 2.0°
Absorption correction: multi-scan (*SADABS*; Sheldrick, 1996)	*h* = −9→9
*T*_min_ = 0.953, *T*_max_ = 0.976	*k* = −17→18
17210 measured reflections	*l* = −22→24

### Refinement

**Table:**

Refinement on *F*^2^	Secondary atom site location: difference Fourier map
Least-squares matrix: full	Hydrogen site location: inferred from neighbouring sites
*R*[*F*^2^ > 2σ(*F*^2^)] = 0.041	H atoms treated by a mixture of independent and constrained refinement
*wR*(*F*^2^) = 0.109	*w* = 1/[σ^2^(*F*_o_^2^) + (0.048*P*)^2^ + 1.0328*P*] where *P* = (*F*_o_^2^ + 2*F*_c_^2^)/3
*S* = 1.08	(Δ/σ)_max_ = 0.030
2225 reflections	Δρ_max_ = 0.40 e Å^−^^3^
167 parameters	Δρ_min_ = −0.42 e Å^−^^3^
0 restraints	Extinction correction: *SHELXL97* (Sheldrick, 2008), Fc^*^=kFc[1+0.001xFc^2^λ^3^/sin(2θ)]^-1/4^
Primary atom site location: structure-invariant direct methods	Extinction coefficient: 0.0062 (8)

### Special details

**Table:**

Geometry. All e.s.d.'s (except the e.s.d. in the dihedral angle between two l.s. planes) are estimated using the full covariance matrix. The cell e.s.d.'s are taken into account individually in the estimation of e.s.d.'s in distances, angles and torsion angles; correlations between e.s.d.'s in cell parameters are only used when they are defined by crystal symmetry. An approximate (isotropic) treatment of cell e.s.d.'s is used for estimating e.s.d.'s involving l.s. planes.
Refinement. Refinement of *F*^2^ against ALL reflections. The weighted *R*-factor *wR* and goodness of fit *S* are based on *F*^2^, conventional *R*-factors *R* are based on *F*, with *F* set to zero for negative *F*^2^. The threshold expression of *F*^2^ > σ(*F*^2^) is used only for calculating *R*-factors(gt) *etc*. and is not relevant to the choice of reflections for refinement. *R*-factors based on *F*^2^ are statistically about twice as large as those based on *F*, and *R*- factors based on ALL data will be even larger.

### Fractional atomic coordinates and isotropic or equivalent isotropic displacement parameters (Å^2^)

**Table:**

	*x*	*y*	*z*	*U*_iso_*/*U*_eq_	
S1	0.24520 (8)	0.67345 (3)	0.06193 (3)	0.0654 (2)	
C6	0.0560 (2)	0.57828 (11)	0.14810 (8)	0.0434 (4)	
N1	0.13723 (18)	0.50268 (9)	0.12517 (7)	0.0460 (4)	
C1	0.0870 (2)	0.66009 (11)	0.12020 (9)	0.0473 (4)	
C8	0.1974 (2)	0.49538 (11)	0.06126 (8)	0.0415 (4)	
O1	0.4535 (2)	0.53800 (12)	−0.16387 (7)	0.0733 (4)	
C12	0.3099 (2)	0.56256 (12)	−0.03694 (9)	0.0480 (4)	
H12	0.3371	0.6129	−0.0601	0.058*	
C11	0.3359 (2)	0.48174 (13)	−0.06591 (8)	0.0488 (4)	
C14	0.1335 (2)	0.42393 (12)	0.16677 (10)	0.0541 (5)	
H14A	0.1398	0.4419	0.2124	0.065*	
H14B	0.2313	0.3894	0.1574	0.065*	
C7	0.2448 (2)	0.56995 (11)	0.02515 (9)	0.0434 (4)	
C2	0.0052 (3)	0.73369 (13)	0.14222 (10)	0.0583 (5)	
H2	0.0247	0.7871	0.1219	0.070*	
C5	−0.0556 (2)	0.57511 (13)	0.20039 (10)	0.0554 (5)	
H5	−0.0786	0.5219	0.2204	0.066*	
C13	0.4132 (3)	0.47568 (17)	−0.13082 (10)	0.0598 (5)	
C9	0.2197 (2)	0.41474 (12)	0.03065 (10)	0.0506 (5)	
H9	0.1869	0.3643	0.0525	0.061*	
C10	0.2888 (2)	0.40786 (13)	−0.03095 (10)	0.0540 (5)	
H10	0.3043	0.3530	−0.0495	0.065*	
C15	−0.0183 (3)	0.36605 (15)	0.15823 (12)	0.0694 (6)	
H15A	−0.1153	0.3976	0.1714	0.104*	
H15B	−0.0068	0.3148	0.1850	0.104*	
H15C	−0.0282	0.3492	0.1129	0.104*	
C4	−0.1325 (3)	0.64997 (17)	0.22290 (11)	0.0677 (6)	
H4	−0.2044	0.6465	0.2585	0.081*	
C3	−0.1046 (3)	0.72895 (16)	0.19376 (11)	0.0679 (6)	
H3	−0.1590	0.7787	0.2085	0.081*	
H13	0.429 (4)	0.4148 (18)	−0.1460 (14)	0.102*	

### Atomic displacement parameters (Å^2^)

**Table:**

	*U*^11^	*U*^22^	*U*^33^	*U*^12^	*U*^13^	*U*^23^
S1	0.0860 (4)	0.0370 (3)	0.0734 (4)	−0.0115 (2)	0.0224 (3)	−0.0011 (2)
C6	0.0406 (9)	0.0475 (10)	0.0421 (9)	0.0007 (7)	−0.0072 (7)	0.0005 (7)
N1	0.0503 (8)	0.0407 (8)	0.0471 (8)	0.0021 (6)	0.0024 (7)	0.0102 (6)
C1	0.0509 (10)	0.0449 (10)	0.0461 (10)	0.0015 (8)	−0.0072 (8)	−0.0011 (8)
C8	0.0400 (8)	0.0382 (9)	0.0464 (9)	−0.0009 (7)	−0.0030 (7)	0.0056 (7)
O1	0.0770 (10)	0.0945 (12)	0.0485 (8)	−0.0086 (9)	0.0078 (7)	−0.0013 (8)
C12	0.0490 (10)	0.0496 (10)	0.0455 (10)	−0.0053 (8)	−0.0021 (8)	0.0086 (8)
C11	0.0427 (10)	0.0585 (12)	0.0453 (10)	0.0012 (8)	−0.0057 (7)	−0.0019 (8)
C14	0.0547 (11)	0.0518 (11)	0.0558 (11)	0.0035 (9)	−0.0008 (9)	0.0213 (8)
C7	0.0452 (10)	0.0373 (9)	0.0476 (10)	−0.0012 (7)	−0.0013 (8)	0.0041 (7)
C2	0.0629 (12)	0.0484 (11)	0.0635 (12)	0.0078 (9)	−0.0119 (10)	−0.0070 (9)
C5	0.0503 (10)	0.0677 (13)	0.0481 (10)	−0.0041 (9)	−0.0001 (9)	0.0016 (9)
C13	0.0505 (11)	0.0781 (15)	0.0508 (11)	0.0003 (11)	−0.0066 (9)	−0.0080 (11)
C9	0.0541 (11)	0.0371 (9)	0.0607 (12)	−0.0007 (8)	0.0000 (9)	0.0055 (8)
C10	0.0545 (11)	0.0474 (11)	0.0600 (12)	0.0036 (9)	−0.0063 (9)	−0.0085 (9)
C15	0.0670 (13)	0.0598 (13)	0.0813 (15)	−0.0072 (10)	0.0038 (11)	0.0263 (11)
C4	0.0539 (12)	0.0902 (17)	0.0589 (12)	0.0050 (11)	0.0047 (10)	−0.0172 (12)
C3	0.0597 (12)	0.0707 (14)	0.0732 (14)	0.0152 (11)	−0.0059 (11)	−0.0206 (12)

### Geometric parameters (Å, °)

**Table:**

S1—C7	1.7538 (18)	C14—H14A	0.9700
S1—C1	1.756 (2)	C14—H14B	0.9700
C6—C5	1.396 (3)	C2—C3	1.376 (3)
C6—C1	1.399 (2)	C2—H2	0.9300
C6—N1	1.411 (2)	C5—C4	1.383 (3)
N1—C8	1.392 (2)	C5—H5	0.9300
N1—C14	1.474 (2)	C13—H13	0.99 (3)
C1—C2	1.382 (3)	C9—C10	1.376 (3)
C8—C9	1.396 (3)	C9—H9	0.9300
C8—C7	1.412 (2)	C10—H10	0.9300
O1—C13	1.212 (3)	C15—H15A	0.9600
C12—C7	1.372 (3)	C15—H15B	0.9600
C12—C11	1.388 (3)	C15—H15C	0.9600
C12—H12	0.9300	C4—C3	1.366 (3)
C11—C10	1.390 (3)	C4—H4	0.9300
C11—C13	1.463 (3)	C3—H3	0.9300
C14—C15	1.524 (3)		
			
C7—S1—C1	100.44 (8)	C3—C2—C1	120.8 (2)
C5—C6—C1	117.13 (17)	C3—C2—H2	119.6
C5—C6—N1	121.61 (16)	C1—C2—H2	119.6
C1—C6—N1	121.24 (16)	C4—C5—C6	120.93 (19)
C8—N1—C6	122.48 (14)	C4—C5—H5	119.5
C8—N1—C14	118.49 (15)	C6—C5—H5	119.5
C6—N1—C14	118.24 (15)	O1—C13—C11	124.4 (2)
C2—C1—C6	120.97 (18)	O1—C13—H13	122.3 (17)
C2—C1—S1	118.17 (15)	C11—C13—H13	113.3 (17)
C6—C1—S1	120.58 (14)	C10—C9—C8	121.78 (17)
N1—C8—C9	122.20 (15)	C10—C9—H9	119.1
N1—C8—C7	121.03 (15)	C8—C9—H9	119.1
C9—C8—C7	116.73 (16)	C9—C10—C11	120.97 (18)
C7—C12—C11	121.50 (17)	C9—C10—H10	119.5
C7—C12—H12	119.3	C11—C10—H10	119.5
C11—C12—H12	119.3	C14—C15—H15A	109.5
C12—C11—C10	117.92 (17)	C14—C15—H15B	109.5
C12—C11—C13	120.29 (19)	H15A—C15—H15B	109.5
C10—C11—C13	121.78 (19)	C14—C15—H15C	109.5
N1—C14—C15	115.30 (16)	H15A—C15—H15C	109.5
N1—C14—H14A	108.4	H15B—C15—H15C	109.5
C15—C14—H14A	108.4	C3—C4—C5	121.2 (2)
N1—C14—H14B	108.4	C3—C4—H4	119.4
C15—C14—H14B	108.4	C5—C4—H4	119.4
H14A—C14—H14B	107.5	C4—C3—C2	118.9 (2)
C12—C7—C8	121.06 (16)	C4—C3—H3	120.5
C12—C7—S1	117.80 (13)	C2—C3—H3	120.5
C8—C7—S1	120.72 (14)		
			
C5—C6—N1—C8	155.35 (17)	N1—C8—C7—C12	177.55 (16)
C1—C6—N1—C8	−26.1 (2)	C9—C8—C7—C12	−0.1 (3)
C5—C6—N1—C14	−14.3 (2)	N1—C8—C7—S1	5.2 (2)
C1—C6—N1—C14	164.21 (16)	C9—C8—C7—S1	−172.52 (13)
C5—C6—C1—C2	−2.4 (3)	C1—S1—C7—C12	157.90 (15)
N1—C6—C1—C2	179.03 (16)	C1—S1—C7—C8	−29.47 (16)
C5—C6—C1—S1	171.37 (14)	C6—C1—C2—C3	2.3 (3)
N1—C6—C1—S1	−7.2 (2)	S1—C1—C2—C3	−171.58 (16)
C7—S1—C1—C2	−155.68 (15)	C1—C6—C5—C4	0.5 (3)
C7—S1—C1—C6	30.42 (16)	N1—C6—C5—C4	179.09 (17)
C6—N1—C8—C9	−155.31 (17)	C12—C11—C13—O1	−2.6 (3)
C14—N1—C8—C9	14.4 (3)	C10—C11—C13—O1	178.44 (19)
C6—N1—C8—C7	27.1 (2)	N1—C8—C9—C10	−175.86 (17)
C14—N1—C8—C7	−163.21 (16)	C7—C8—C9—C10	1.8 (3)
C7—C12—C11—C10	2.1 (3)	C8—C9—C10—C11	−1.5 (3)
C7—C12—C11—C13	−176.89 (17)	C12—C11—C10—C9	−0.5 (3)
C8—N1—C14—C15	−84.5 (2)	C13—C11—C10—C9	178.53 (18)
C6—N1—C14—C15	85.6 (2)	C6—C5—C4—C3	1.5 (3)
C11—C12—C7—C8	−1.8 (3)	C5—C4—C3—C2	−1.6 (3)
C11—C12—C7—S1	170.76 (14)	C1—C2—C3—C4	−0.3 (3)

### Hydrogen-bond geometry (Å, °)

**Table:**

*D*—H···*A*	*D*—H	H···*A*	*D*···*A*	*D*—H···*A*
C14—H14A···O1^i^	0.97	2.64	3.563 (3)	158.

Symmetry codes: (i) −*x*+1/2, −*y*+1, *z*+1/2.

## References

[bb1] Bruker (2002). *SMART* and *SAINT* Bruker AXS Inc., Madison, Wisconsin, USA.

[bb2] Chu, S. S. C. & Van der Helm, D. (1975). *Acta Cryst.* B**31**, 1179–1183.

[bb3] Hdii, F., Reboul, J.-P., Barbe, J., Siri, D. & Pèpe, G. (1998). *Acta Cryst.* C**54**, 1151–1152.

[bb4] Li, D. M., Hu, R. T., Zhou, W., Sun, P. P., Kan, Y. H., Tian, Y. P., Zhou, H. P., Wu, J. Y., Tao, X. T. & Jiang, M. H. (2009*b*). *Eur. J. Inorg. Chem.* pp. 2664–2672.

[bb5] Li, D. M., Lv, L. F., Sun, P. P., Zhou, W., Wang, P., Wu, J. Y., Kan, Y. H., Zhou, H. P. & Tian, Y. P. (2009*a*). Dyes Pigments, **83**, 180–186.

[bb6] Sheldrick, G. M. (1996). *SADABS* University of Göttingen, Germany.

[bb7] Sheldrick, G. M. (2008). *Acta Cryst.* A**64**, 112–122. 10.1107/S0108767307043930 18156677

